# Appendiceal Endometriosis Presenting As Chronic Appendicitis: A Case Report

**DOI:** 10.7759/cureus.37825

**Published:** 2023-04-19

**Authors:** Sarah Klein, RaeAnn Tourangeau-Young, Alejandro Biglione

**Affiliations:** 1 Internal Medicine, Nova Southeastern University Dr. Kiran C. Patel College of Osteopathic Medicine, Clearwater, USA; 2 Internal Medicine, Wellington Regional Medical Center, Wellington, USA

**Keywords:** endometriosis surgery, dysmenorrhea endometriosis, laparoscopic surgery for endometriosis, chronic appendicitis, endometriosis and chronic pelvic pain, appendiceal endometriosis

## Abstract

The origin of endometriosis has multiple theories, with controversy over which may demonstrate the prominent pathophysiology. The most common extra-pelvic organ system affected by endometriosis is the gastrointestinal tract. Gastrointestinal endometriosis (GE) accounts for 3 to 37% of all endometriosis cases, and appendiceal endometriosis is present in around 3% of GE cases, therefore constituting less than 1% of all endometriosis cases.

In this report, we present a 24-year-old female with a past medical history significant for endometriosis status post two excisional laparoscopies who presented with eight months’ duration of right lower quadrant pain, constant and stabbing, with rebound tenderness. Appendectomy and histopathology demonstrated focal endometriosis, diffuse serosal fibrovascular adhesions involving the appendiceal serosa/subserosa, as well as a dilated lumen filled with hemorrhagic content.

When the appendix is not considered in endometriosis pathology, patients are at increased risk for unresolved pain and further laparoscopic procedures. Prophylactic appendectomy appears to be a worthwhile consideration in patients with chronic pelvic pain, given the high frequency of appendiceal pathology.

## Introduction

Endometriosis occurs when endometrial glands and stroma are found outside the uterine cavity or musculature [1]. The origin of the disease is still undetermined and there is controversy over leading theories of its origin. These theories include Sampson’s theory, which states that endometrial lesions are implanted into the pelvic cavity through retrograde menstruation; Bronsen and Benagiano's theory, which suggests that endometriosis lesions originate from retrograde bleeding that occurs due to neonatal hormonal deprivation; a theory that suggests that Mullerian duct embryonic cells persist in ectopic locations; and finally, yet another theory that suggests a genetic risk for endometriosis [2]. As described, the origin of endometriosis has multiple theories, with controversy over which theory may demonstrate the prominent pathophysiology.

Current options for medical management of endometriosis symptoms include non-steroidal anti-inflammatory drugs (NSAIDs), combined oral contraceptives, progestins, gonadotropin-releasing hormone agonists, gonadotropin-releasing hormone antagonists, and aromatase inhibitors [3]. However, these medical management options only work to suppress ovarian function and are not curative for endometriosis. Surgical management of endometriosis includes ablation and excision of endometriosis lesions as well as neural ablation and resection for pain management. Medical versus surgical management of endometriosis is a broad and multifactorial decision, and currently, neither can be recommended over the other [4].

The most common extra-pelvic organ system affected by endometriosis is the gastrointestinal tract. Of the gastrointestinal tract, the sigmoid colon is the most commonly involved, followed by the rectum, ileum, appendix, and cecum [5]. Appendiceal endometriosis (AE) was first described in 1860 [6]. Gastrointestinal endometriosis (GE) accounts for 3 to 37% of all endometriosis cases, and AE is present in around 3% of GE cases, therefore constituting less than 1% of all endometriosis cases [7,8]. For many women, receiving a diagnosis of endometriosis is a long process plagued by misdiagnoses, barriers to care, and psychosocial impacts. Common barriers to proper diagnosis and treatment include the wide variety and severity of symptoms present in patients with endometriosis, as well as the diagnostic gold standard of an invasive surgical laparoscopic procedure. These barriers have posed a well-established delay of four to 11 years from first symptom onset to surgical diagnosis [9]. Apart from the controversy in the pathogenesis of endometriosis, the disease carries multiple other factors that complicate the diagnosis, treatment, and understanding of this disease as a whole.

## Case presentation

We are presenting a 24-year-old nulliparous female with a significant past medical history of endometriosis who presented to the clinic with right lower quadrant pain of eight-month duration. The pain was not correlated with the patient’s menstrual cycles and occurred nearly every day. On presentation, symptoms included bloating, a sense of heaviness in the pelvic region that was worse at the end of each day, and bowel habit changes, including both diarrhea and constipation. The patient also described a constant, dull, aching pain that was frequently interrupted by bouts of severe pain that was stabbing in nature and accompanied by nausea. The patient reported that her pain was not relieved by non-steroidal anti-inflammatory drugs (NSAIDs) and that a heating pad and rest were the only treatments that gave her relief. The patient takes norethindrone 5mg daily for suppression of endometriosis as well as contraception. The patient has a history of two laparoscopic procedures for diagnosis of endometriosis, excision of endometrioma, endometriotic lesions, and adhesions in locations including the bilateral ovaries and the bladder wall, uterine wall, and abdominal wall. On physical examination, the patient has right lower quadrant hypertonicity, guarding, and rebound tenderness. The pelvic exam was nonsignificant, and laboratory studies and ultrasound imaging failed to reveal any acute or abnormal pathology.

**Figure 1:**
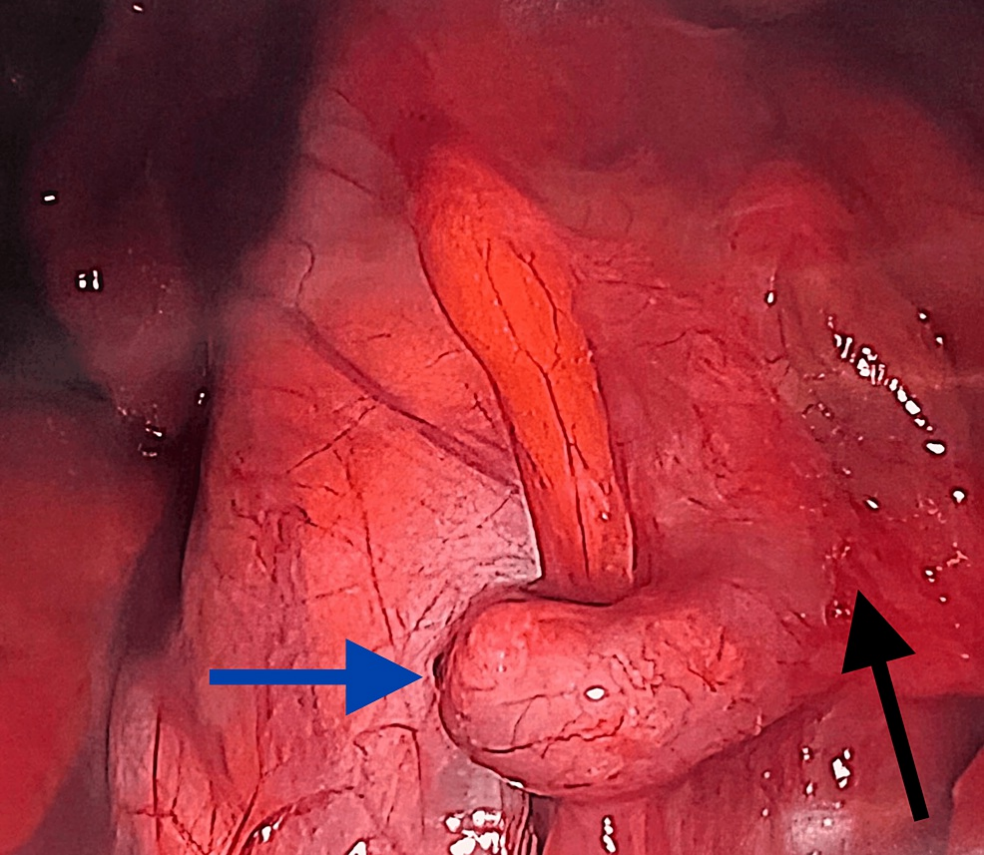
Laparoscopy imaging showing a mildly dilated appendix (blue arrow) with significant scar tissue around the appendix and the cecum (black arrow).

**Figure 2:**
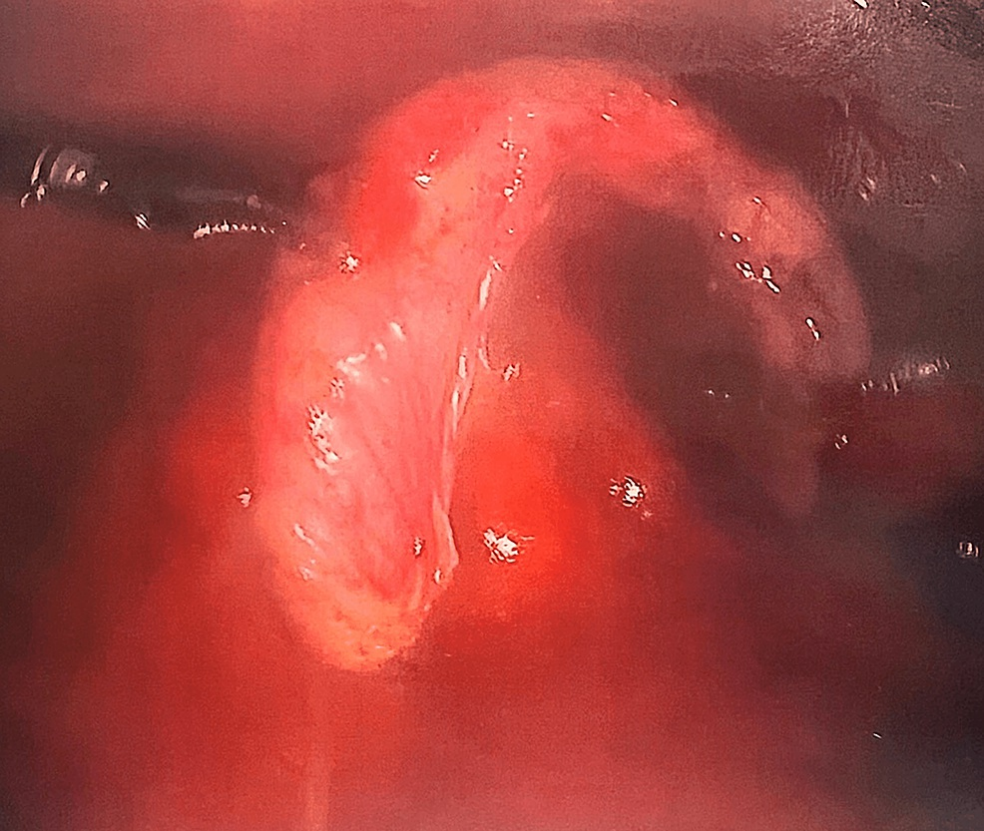
The appendix post-appendectomy

A conservative treatment consisting of norethindrone 5mg daily failed to improve the patient’s symptoms. After a discussion of different treatment options and their risks and benefits, the patient decided on laparoscopic exploration for recurrent endometriotic lesions. During the laparoscopic procedure, no recurrent endometriotic lesions were found on the bilateral ovaries, uterus, bladder, or abdominal wall. The appendix was found to be mildly dilated (Figure 1, blue arrow) with significant scar tissue around the appendix and the cecum (Figure 1, black arrow). The appendix was excised, and histopathology demonstrated focal endometriosis, diffuse serosal fibrovascular adhesions involving the appendiceal serosa and subserosa, as well as a dilated lumen filled with hemorrhagic content (Figure 2). Since the procedure, the patient reports a complete resolution of symptoms. The patient continues on norethindrone 5mg daily for suppression of endometriosis recurrence and contraception.

## Discussion

While rare, appendiceal endometriosis should be considered in the differential diagnosis of females with chronic pelvic pain. Patients are already facing obstacles to diagnosis, including the variety in symptomatology, controversy in pathogenesis and medical treatment, and the need for a laparoscopic procedure for a definitive diagnosis. In addition to these obstacles, the great variety in the gross appearance of endometriotic lesions presents a further challenge for complete surgical excision. While typical endometriotic lesions have a blue-black pigment, there are non-pigmented lesions that can impede the gross and conclusive recognition of endometriosis during the laparoscopic procedure. These lesions include but are not limited to, white opacified peritoneum, red flamelike lesions, glandular lesions, and yellow-brown peritoneal patches [10]. Even when not grossly visualized in the appendix during a laparoscopic procedure, endometriotic lesions can still be present within the appendiceal tissue. In a study done by Jocko et al., of 71 appendices that had appeared normal on gross visualization during laparoscopy but were removed, 44% had positive pathology, regardless of diagnosis [11]. In a study performed by Lyons et al., 154 out of 190 females undergoing laparoscopic surgery for pelvic pain had an abnormal pathology of the appendix. This study endorsed the idea that prophylactic appendectomy appears to be a worthwhile consideration in patients with chronic pelvic pain, given the high frequency of appendiceal pathology [12].

## Conclusions

Endometriosis is a quite complicated and poorly understood disease, and for patients already dealing with painful symptomatology, psychosocial impacts, and a delay in diagnosis, the variety of pathologic presentation presents a barrier to patients getting adequate and timely treatment and resolution of symptoms. When the appendix is not considered in disease pathology, patients are at increased risk of unresolved pain and, thus, further laparoscopic procedures. Therefore, a prophylactic appendectomy should be considered in patients with chronic pelvic pain who are already undergoing laparoscopy. More research is needed to determine the benefits and risks of prophylactic appendectomy in chronic pelvic pain patients.
